# Are Long-Range Structural Correlations Behind the Aggregration Phenomena of Polyglutamine Diseases?

**DOI:** 10.1371/journal.pcbi.1002501

**Published:** 2012-04-26

**Authors:** Mahmoud Moradi, Volodymyr Babin, Christopher Roland, Celeste Sagui

**Affiliations:** Center for High Performance Simulations (CHiPS) and Department of Physics, North Carolina State University, Raleigh, North Carolina, United States of America; University of Houston, United States of America

## Abstract

We have characterized the conformational ensembles of polyglutamine 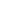 peptides of various lengths 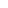 (ranging from 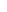 to 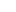), both with and without the presence of a C-terminal polyproline hexapeptide. For this, we used state-of-the-art molecular dynamics simulations combined with a novel statistical analysis to characterize the various properties of the backbone dihedral angles and secondary structural motifs of the glutamine residues. For 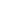 (*i.e.*, just above the pathological length 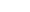 for Huntington's disease), the equilibrium conformations of the monomer consist primarily of disordered, compact structures with non-negligible 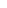-helical and turn content. We also observed a relatively small population of extended structures suitable for forming aggregates including 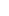
- and 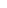-strands, and 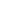
- and 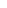-hairpins. Most importantly, for 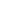 we find that there exists a *long-range* correlation (ranging for at least 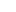 residues) among the backbone dihedral angles of the Q residues. For polyglutamine peptides below the pathological length, the population of the extended strands and hairpins is considerably smaller, and the correlations are short-range (at most 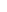 residues apart). Adding a C-terminal hexaproline to 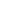 suppresses both the population of these rare motifs and the *long-range* correlation of the dihedral angles. We argue that the long-range correlation of the polyglutamine homopeptide, along with the presence of these rare motifs, could be responsible for its aggregation phenomena.

## Author Summary

Nine neurodegenerative diseases are caused by polyglutamine (polyQ) expansions greater than a given threshold in proteins with little or no homology except for the polyQ regions. The diseases all share a common feature: the formation of polyQ aggregates and eventual neuronal death. Using molecular dynamics simulations, we have explored the conformations of polyQ peptides. Results indicate that for  peptides (*i.e.*, just above the pathological length for Hungtington's disease), the equilibrium conformations were found to consist primarily of disordered, compact structures with a non-negligible -helical and turn content. We also observed a small population of extended structures suitable for forming aggregates. For peptides below the pathological length, the population of these structures was found to be considerably lower. For longer  peptides, we found evidence for long-range correlations among the dihedral angles. This correlation turns out to be short-range for the smaller polyQ peptides, and is suppressed (along with the extended structural motifs) when a C-terminal polyproline tail is added to the peptides. We believe that the existence of these long-range correlations in above-threshold polyQ peptides, along with the presence of rare motifs, could be responsible for the experimentally observed aggregation phenomena associated with polyQ diseases.

## Introduction

Polyglutamine (polyQ) diseases involve a set of nine late-onset progressive neurodegenerative diseases caused by the expansion of CAG triplet sequence repeats [1]. These repeats result in the transcription of proteins with abnormally long polyQ inserts. When these inserts expand beyond a normal repeat length, the affected proteins form toxic aggregates [2] leading to neuronal death. PolyQ aggregation takes place through a complex multistage process involving transient and metastable structures that occur before, or simultaneously, with fibril formation [3]–[9]. Experimental findings suggest that the therapeutic target for polyQ diseases should be the soluble oligomeric intermediates, or the conformational transitions that lead to them [9], [10], and not the insoluble ordered fibrils. These findings, common to all amyloid diseases [11], have spurred efforts to understand the structural attributes of soluble oligomers and amyloidogenic precursors.

The free energy landscapes of polyQ aggregates display countless minima of similar depth that correspond to a great variety of metastable and/or glassy states. The aggregation kinetics of pure polyQ have been described as a nucleation-growth polymerization process [4]–[6], [12], where soluble expanded glutamine requires a considerable time lag for the creation of a critical nucleus, which then readily converts into a sheet in the presence of a template [13]. However, the “time lag” seems to properly be associated with the formation of the fully aggregated precipitates, since soluble aggregates – sometimes called “protofibrils” – that form during the putative lag phase have been reported [14], [15]. The variety of polyQ soluble and insoluble aggregates might correlate with the conformational flexibility of monomeric (non-aggregate single-chain) polyQ regions, which are influenced by the conformations of neighboring protein regions [4], [16]–[18]. One striking example of this conformational wealth – and still a source of controversy– is given by the polyQ expansion in the N-terminal of the huntingtin protein that is encoded in the exon 1 (EX1) of the gene. The N-terminal amino acid sequence consists of a seventeen, mixed residue sequence, the polyQ region of variable length, two polyproline regions of 11 and 10 residues separated by a region of mixed residues, and a C-terminal sequence. Toxicity develops after the polyQ expansion exceeds a threshold of approximately 36 repeats, leading to Huntington's disease. The flanking sequences have been shown to play a structural role in polyQ sequences, both in synthetic and natural peptides, and both in monomeric or aggregate form [4], [16], [17], [19]. In particular, a polyproline (polyP) region immediately adjacent to the C-terminal of a polyQ region has been shown to affect the conformation of the polyQ region; the resulting conformations depend on the lengths of both the polyQ and polyP sequences [16], [17], [20], [21].

In this work, we set out to obtain a conceptual and quantitative understanding of the role played by a polyP sequence that is placed at the C-terminal of a polyQ peptide, which is relevant for the understanding of the behavior of the EX1 segment in the huntingtin protein. Sedimentation aggregation kinetics experiments [17] show that the introduction of a 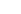 sequence C-terminal to polyQ in synthetic peptides decreases both the rate of formation and the apparent stability of the associated aggregates. The polyP sequence can be trimmed to 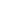 without altering the suppression effect, but a 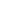 sequence is ineffective. There are no effects when the polyP sequences are attached to the N-terminal or via a side-chain tether [17]. These experiments were complemented with CD spectra for monomeric peptides, where the presence of polyP at the C-terminal of 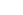 showed remarkable changes in the spectra. Analysis of their data led the authors to propose that addition of the C-terminal 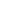 sequence does not alter the aggregation mechanism, which is nuclefated growth by monomer addition with a critical nucleus of 1 monomer (for 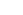), but destabilizes both the 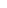-helical and the (still unknown) aggregation-competent conformations of the monomer. These experimental results were unexpected: although a single proline residue interrupting an amyloidogenic sequence can decrease the propensity of that sequence to aggregate [22], [23], Pro replacements in amyloidogenic sequences placed in turns or disordered regions do not alter the aggregate core [23].

Here, we consider monomeric polyQ and polyQ-polyP chains, and quantify changes brought about in the conformations of the polyQ sequences by the addition of the polyP sequences at their C-terminal. In order to assess these changes, one must first characterize the conformation of pure monomeric polyQ in water. Wildly diverse conformations have been postulated experimentally for monomeric polyQ, including a totally random coil, 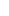-sheet, 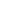-helix, and PPII structures. At present there is growing experimental evidence that single polyQ chains are mainly disordered [6], [13]–[15]. The solvated polyQ disorder, however, is different from a total random coil or a protein denatured state. In particular, atomic X-ray experiments [18] show that single chains of polyQ (in the presence of flanking sequences) present isolated elements of 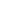-helix, random coil and extended loop. Single-molecule force-clamp techniques were used to probe the mechanical behavior of polyQ chains of varying lengths spanning normal and diseased polyQ expansions [24]. Under the application of force, no extension was observed for any of the polyQ constructs. Further analysis led the authors to propose that polyQ chains collapse to form a heterogeneous ensemble of globular conformations that are mechanically stable.

Simulations results for the monomer conformation have also been contradictory [25]–[31]. It is interesting that in the search for soluble prefibrillar intermediates, an 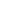-sheet was proposed to play a role in polyQ toxicity [32], [33]. In these molecular dynamics simulations, polyQ monomers of various lengths were found to display transient 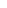-strands of four residues or less. The authors proposed that fibril formation in polyQ may proceed through 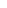 strands intermediates [33]. More recently, a molecular dynamics study of hexamers of 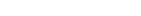 in explicit water showed that 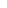-sheet aggregates are very stable (more stable than 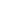-sheets) [34]. These results strongly support the idea that 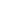-sheet may either be a stable, a metastable, or at least a long-lived transient, secondary structure of polyQ aggregates. Coming back to the monomeric polyQ conformation, further simulation evidence [35]–[38] supports the experimental findings that monomeric polyglutamine of various lengths is a disordered statistical coil in solution. The disorder is inherently different from that of denatured proteins and the average compactness and magnitude of conformational fluctuations increase with chain length [35]. In addition, the coils may present considerable 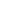-helical content [38], but there are acute entropic bottlenecks for the formation of 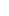-sheets.

The molecular dynamics results presented here for single polyQ and polyQ-PolyP chains consisting of 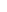, 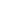, 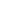, 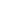, 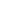, and 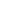 glutamine residues are in qualitative agreement with the experimental and simulation results mentioned above: polyQ is primarily disordered, with non-negligible 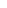-helical content and a small population of other secondary structures including both 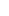 and 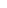 strands. The addition of polyP reduces the population of the 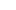 region of Ramachandran plot [39], and increases the population of 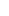 and PPII Ramachandran regions for all PolyQ lengths. If one considers secondary structure motifs (i.e., hydrogen-bonds patterns in addition to dihedral angles), the addition of the polyP segment increases the populations of the PPII helices and turns, and decreases the 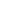-helical content of all peptides but 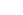 (which may have a protective effect against aggregation, as discussed later). The addition of polyP does not change the average radius of gyration of polyQ, but changes the radius of gyration distribution function for 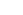, that becomes dependent on the prolyl bond isomerization state. Most importantly, the addition of polyP decreases the population of small 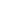 and 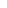 strands, and 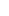 and 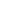 hairpins.

Since the extended strands and hairpins in both 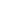 and 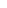 forms are found only in a small fraction of the structures, we used a novel statistical measure based on the odds ratio construction [40] to quantify to study the secondary structural propensities [41], [42], thereby learning about the possibility of the growth of such secondary structures under nucleation conditions. This study, also supported by more conventional linear correlation analysis, provides evidence that among all the peptides studied here, only 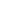 exhibits a *long-range* correlation between all glutamine residue pairs that favors formation of both 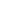 and 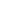-strands. This correlation is suppressed by the addition of only six proline residues to the C-terminal of the peptide, which suggests a mechanism in which nucleation starts at these scarcely populated secondary structures (mainly 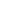, 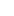, 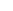 and 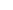 strands, as well as 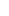-hairpins and 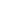-hairpins) and can only spread through positive correlations in polyQ peptides of approximately 40 residues or longer.

This paper is organized as follows. The *Methods* section details our simulation methodology and analysis. Specifically, we discuss the generalized Replica Exchange scheme used here for enhanced sampling, the simulation details, our clustering techniques to identify the Ramachandran regions and the secondary structural motifs, and the odds ratio construction, used here to study the correlations between residues. In the *Results* section, we present our results with a focus on a statistical analysis of the equilibrium conformations based on (i) Ramachandran regions (ii) secondary structure (iii) correlation analysis and (iv) radius of gyration. A discussion of our results and a short summary of this work is given in the last section.

## Methods

In this section, we briefly describe the generalized replica exchange molecular dynamics [41]–[44] approach used to generate the equilibrium conformations. In addition, we describe our quantification of the secondary structural content, and review the odds ratio [40] construction for correlations between residues. For a more detailed description of our simulation methods and the clustering approach used to classify the secondary structure motifs of the peptides, please see the Supporting Information section.

### Sampling Protocol

Room temperature, regular molecular dynamics (MD) simulations are often too computationally limited to carry out a full sampling of the conformational space of a biomolecular system and generate a reliable statistical ensemble. Thus, in order to deal with the sampling issue, we make use of a replica exchange scheme [43], [45]. In the replica exhange molecular dynamics (REMD) [43], [46] method, one considers several replicas of a system subject to some sort of ergodic dynamics based on different Hamiltonians, and attempts to exchange the trajectories of these replicas at a predetermined rate to increase the barrier crossing rates (*i.e.*, decrease the ergodic time scale). One possibility is to successively increase the temperatures of the replicas [46]. This method, known as parallel tempering, is here referred to as Temperature REMD (T-REMD). Another possibility [43] is to construct the replicas by adding a biasing potential to the original Hamiltonian that acts on some collective variable that describes the slow modes of the system that need “acceleration”. This method can be referred to as Hamiltonian REMD (H-REMD). In practice, T-REMD is used to promote the barrier crossing events in a generic way but the use of H-REMD allows one to directly focus on specific slow modes of the system, such as the cis-trans isomerization of proline amino acids which involves a barrier of 10 to 20 Kcal/mol [47]. A combination of the two methods, known as Hamiltonian-Temperature REMD (HT-REMD) [41]–[44] provides for a practical way to reduce the computational costs associated with REMD sampling, since it facilitates the sampling by both means.

In this work, we used the T-REMD and HT-REMD methods for polyQ and polyQ-polyP peptides, respectively. In the T-REMD method, one replica runs at room temperature and the rest of the replicas run at higher temperatures. Care must be taken with respect to the choice of the number of replicas and their temperatures. The performance of the setting can be checked by monitoring the exchange rate between the neighboring replicas (*i.e.*, with closest temperatures) as well as the ergodic time scale of the “hottest” replica. The equilibrium conformational ensemble is then generated by taking the structures at a predetermined rate from the trajectory of the replica at the lowest (room) temperature.

In the HT-REMD method, the replicas have different biasing potentials. The biasing potential is usually described in terms of a *collective variable* 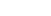, defined as a smooth function of the atomic positions 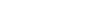. The corresponding free energy or potential of mean force (PMF) [48], 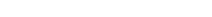 (where the angular brackets denote the equilibrium ensemble average), provides for an ideal biasing potential. Indeed, if the biasing potential is exactly 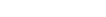, then the probabilities of different values of the collective variable would all be equal, since there are no barriers present. Although the true free energy 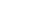 is typically unknown in advance, a roughly approximate 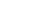 is often sufficient to improve the sampling considerably in an H-REMD or HT-REMD setting. Such free energies can be computed in a variety of ways [48]. For the polyQ-polyP systems, some of the slow modes originate in the cis-trans isomerization of the prolyl bonds, that occur when polyproline is in solution. We have recently carried out extensive work on proline-rich systems [41], [42], [44], [47], [49] and can take advantage of the free energy profiles previously obtained for polyproline of various lengths [44], calculated using the Adaptively Biased Molecular Dynamics (ABMD) [50], [51] method. The ABMD method is an umbrella sampling method with a time-dependent biasing potential, which can be used in conjunction with the REMD protocol, by combining different collective variables and/or temperatures on a per-replica basis [43], [50]. Currently, the ABMD method has been implemented into the AMBER v.10,11 simulation package [52]. Details of the calculation of the polyproline potentials are given elsewhere [41], [42], [44], [47].

The HT-REMD simulations proceeded in several stages. We recycled the previously computed free energies associated with a collective variable that “captures” the cis-trans transitions of the prolyl bonds of polyproline peptides of different lengths in implicit water at different temperatures.

The collective variable used for these calculations is defined based on the backbone dihedral angle 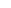 of prolyl bonds, 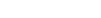 (here sum runs over all the prolyl bonds 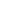). The dihedral angle 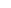 takes the values around 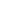 and 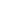 for cis and trans conformations, therefore 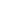 can “capture” different patterns of the cis/trans conformations in any proline-containg peptide. The biasing potentials, transfered from our previous calculations were then refined for the polyQ-polyP peptides using similar simulation settings. Next, several additional replicas running at the lowest temperature 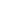 were introduced into the setup. One of these replicas is completely unbiased, and therefore samples the Boltzmann distribution at 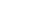. The other replicas, also at 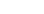, are subject to a reduced bias (*i.e.*, these biasing potentials are scaled down by a constant factor). The purpose of these “proxy” replicas is to ensure adequate exchange rates between the conformations, and thereby enhance the mixing [43]. Data was then taken from the unbiased replica at a suitable, predetermined rate.

### Simulation Details

Simulations were carried out for the peptides with sequence 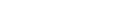 (denoted as 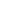) and 
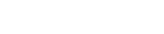
 (denoted as 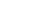). These peptides include 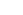, 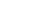, 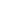, 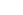, 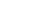, 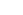, 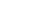, 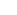, 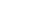, 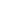, and 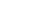. In each case, we refer to the 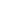 glutamine and 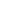 proline residues as 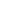 and 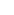, respectively. The simulations were carried out using the AMBER [52] simulation package with the ff99SB version of the Cornell *et al* force field [53] with an implicit water model based on the Generalized Born approximation (GB) [54], [55] including the surface area contributions computed using the LCPO model [56] (GB/SA). For more simulation details, our implementation of the REMD scheme and a discussion of convergence issues, please see the Supporting Information (Text S1).

### Secondary Structure

**Figure 1:**
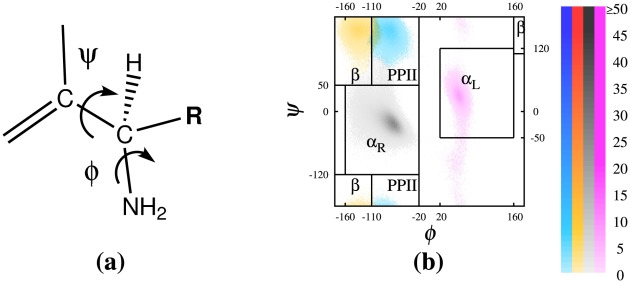
(a) Schematic of amino acid backbone dihedrals 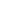
 **and** 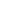 **, and (b) a corresponding Ramachandran plot.** In a typical Ramachandran plot of a glutamine residue, each pixel represents a 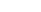 bin, whose intensity represents its relative population, ranging from 1,2,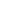,9, and 10 or more conformations, sampled in our simulations. Blue, yellow, grey, and pink clusters identify PPII, 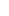, 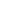, and 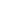 regions, respectively.

We used the (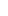, 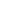) dihedral angles (see Fig. 1 for their definition) to identify different regions [57] of the Ramachandran map [39]. Table 1 provides the corresponding definition for these regions. Although this delineates clear regions for the dihedrals of most residues, it turns out that the populations may overlap around the borders. In order to handle this situation, we used a clustering technique as explained in the Supporting Information (Text S1) to classify the conformations, rather than strictly enforcing the sharp boundaries between the defined regions.

**Table 1:** Secondary structure definitions.

Ramachandran regions	
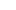	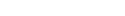 , 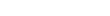
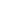	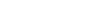 , 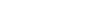
PPII	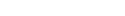 ,( 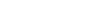 or 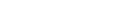 )
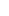	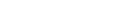 ,( 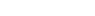 or 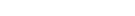 ) or 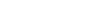 , 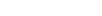

For a detailed description see *Methods*.

Although the backbone dihedral angles of all the residues forming a right-handed 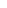-helix fall into the 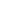 region of Ramachandran map, many of the residues in this region do not actually form 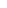-helices. As a matter of fact, several other secondary structural motifs, such as 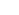 and 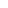 helices as well as random coil and turn are characterized by or may involve backbone dihedral angles falling in the same region. An interesting example is provided by polyglutamine itself. It has been suggested recently [32]–[34] that an 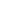-sheet, whose backbone dihedral angles alternate between the 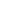 and 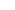 helical regions, can be a stable, metastable, or at least a long-lived transient secondary structure in oligomers.

In general, for a residue to be considered to belong to a given secondary structure, it is not enough to identify the Ramachandran region of its dihedral angles. Thus, we used the secondary structure prediction program DSSP [58], [59] that uses not only the backbone diheral angles, but also the inter-residual hydrogen bonding as well as the relative position of the C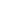 atoms to identify secondary structural motifs. For our peptides, the DSSP secondary structures with highest probabilities were: (i) helices, including 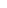 and 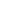 types, (ii) turns, including H-bonded turns and bends, (iii) coils. There are also isolated residues involved in 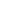 bridges and extended strands, participating in the 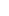 ladders with small probabilities. Since DSSP does not specifically identify isolated 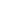 or 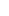 strands (*i.e.*, strands not H-bonded to another strand of their type) or 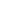 hairpins, we used a combination of H-bonding results from DSSP analysis and the Ramachandran regions from the clustering analysis to define 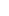 and 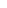 strands and hairpins. A 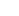 strand is defined here as at least 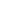 adjacent residues all falling into the 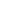 region of Ramachandran plot. A 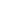 strand is referred to as isolated if none of its 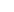 residues is H-bonded. A 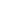 hairpin is defined as two adjacent 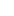 strands with a turn in between and at least one H-bond between the two strands. The turn between the two strands of a hairpin could be H-bonded or not and is of any length but it has to have the geometrical form of a turn, (*i.e.*, identified as bend by DSSP). Each of the two strands has at least three adjacent residues in 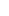 region to ensure the structure is relatively extended. At least one of these three 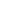 residues are H-bonded to another 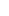 residue in the other strand. We define an 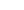 repeat as two adjacent residues, whose backbone dihedral angles alternate between 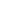 and 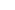 regardless of the order (*i.e.*, this includes both 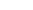 and 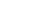). An 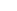 strand is formed from 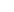 adjacent residues, involving 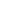 alternating 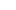 and 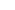 repeats. In this definition, an 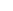 strand is either 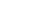 or 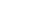 and an 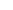 strand is either 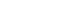 or 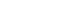 but not 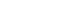. An isolated 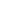 strand is defined as an 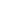 strand not H-bonded to another strand, and the 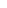 hairpin is defined as two adjacent 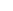 strands with a turn in between and at least one H-bond between the two strands, similar to the 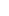 hairpin. Another relatively extended secondary structure is PPII that is defined here as adjacent residues whose dihedral angles fall into the PPII region of Ramachandran plot. A PPII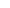 structure, is defined as a structure having 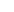 adjacent PPII residues. A summary of these secondary structures is given in Table 1.

Finally, we determined the type of turn from both the DSSP analysis and our Ramachandran region clustering analysis. DSSP distinguishes between H-bonded turns and geometrical bends that do not involve any H-bonding. The DSSP analysis can be also used to identify 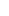 and 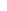 types based on the number of residues involved, which is 4 and 3 respectively. The dihedral angles of the two middle residues of 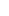 turns (*i.e.* the second and the third residues) can be used to partition 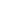 turns into more types such as I, I′, II, II′, etc. but we will only consider type I-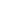 that involves an 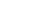 sequence and the “other” type 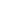 turns that involve other combinations of dihedral angles. Since the population of “other” combinations is relative small, we group these all together.

### Odds Ratio

To quantify how the secondary structures of Gln residues influence each other we made use of the odds ratio (OR) construction [40]–[42]. The OR is a descriptive statistic that measures the strength of association, or non-independence, between two binary values. The OR is defined for two binary random variables (denoted as 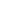 and 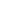) as:
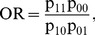
(1)where 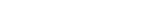 is the joint probability of the 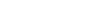 event (with 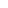 and 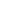 taking on binary values of 0 and 1). For the purposes of this study, we can think of 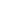 and 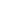 as being some characteristic properties describing the conformations of different residues. For example, the variables could be assigned values of 1 or 0 depending on whether the backbone dihedral angles of corresponding residue falls into the 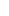 region of Ramachandran plot or not. We denote this definition of OR as OR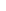. Similarly one can define 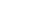 based on the involvement of residues in 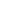 repeats. In this case, to define the 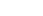 of two given residues 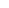 and 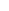, the probabilities 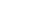 are defined such that the variables 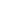 and 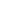 take the values 1 or 0 depending on whether or not the corresponding residue is involved in an 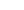 repeat as defined in the last subsection. For instance, 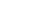 if and only if residue 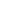 either is in the 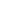 region and is neighboring a residue in the 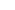 region, or it is in the 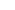 region and is neighboring a residue in the 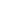 region. Note that in general, to calculate the 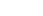 of two residues, dihedral angles of not only the two residues but also their neighbors are needed, *i.e.*, up to 6 residues could be involved.

The usefulness of the OR in quantifying the influence of one binary random variable upon another can be readily seen. If the two variables are statistically independent, then 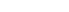 so that 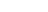. In the opposite extreme case of 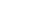 (complete dependence) both 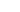 and 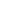 are zero, and the OR is infinite. Similarly, for 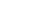 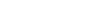 rendering 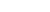. To summarize, an OR of unity indicates that the values of 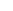 are equally likely for both values of 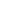 (*i.e.*, 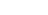, 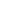 and 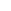 are therefore independent); an OR greater than unity indicates that 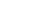 is more likely when 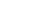 (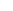 and 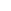 are positively correlated), while an OR less than unity indicates that 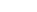 is more likely when 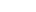 (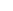 and 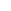 are negatively correlated).

It is convenient to recast the log of the OR in terms of free energy language. If one expresses the probability of the 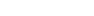 events in terms of a free energy 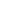:
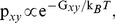
(2)then the ratio of probabilities 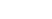 translates into a free energy difference:
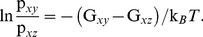
(3)Clearly, the logarithm of the OR then maps onto the difference of those differences, *i.e.*,
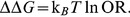
(4)For the case of statistically independent properties, 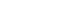; otherwise, this quantity takes on either positive or negative values, whose magnitude depends on the mutual dependence between the two variables. The standard error in its asymptotic approximation is:
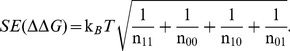
(5)in which 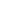 is the total number of independent 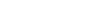 events sampled. While this development may be perceived as purely formal, the use of an OR analysis couched in terms of free energy language provides for a useful and intuitive measure of the inter-residual correlations, as has been illustrated before [41], [42].

In this work, our OR-based correlation analysis is supported by the conventional linear correlation analysis. We have used the correlation coefficient (also know as cross-correlation or Pearson correlation) of 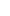 dihedral angles of glutamine residues to measure the correlation of glutamine residues in different situations. We emphasize that in the context of secondary structural propensities, the odds ratio analysis is more powerful than the correlation coefficient since it eliminates the noise associated with the dihedral angles. This noise may dominate the linear correlation results such that even substantial correlations may be completely ignored. The OR-based correlation analysis, combined with the clustering technique explained here takes into account both nonlinearity and multivariate components of amino acid correlations in a peptide chain, although in some particular cases a conventional univariate linear correlation may reveal a correlation as we will report in the results. In the context of this paper, the multivariate component is particularly evident when the correlation of 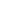 repeats is considered, since this may involve 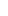 and 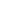 angles of up to six residues for each single odds ratio calculation.

## Results

We generated 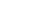 equilibrium structures of the 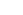 and 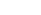 peptides, 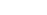 structures of 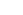, 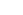, and 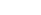, and 10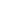 structures of 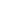, 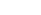, 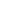, 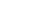, 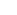, and 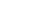 peptides at 300 K to compute the probabilities of different secondary structural motifs and thereby characterize the conformational ensemble of these peptides.

Here, we present our results in terms of (i) the regions of the Ramachandran map occupied by each individual glutamine residue, (ii) the secondary structures identified based not only by the backbone dihedral angles but also by the inter-residual hydrogen bonds and positions of the 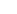 atoms, (iii) a correlation analysis on the dihedral angles of glutamine residues, and (iv) the ensemble distribution of the radius of gyration, describing the overall compactness of the structures. Figures 1–8 (and Figures S1, S2, S3) and Tables 2–3 (and Table S1) summarize these results.

**Figure 2 pcbi-1002501-g002:**
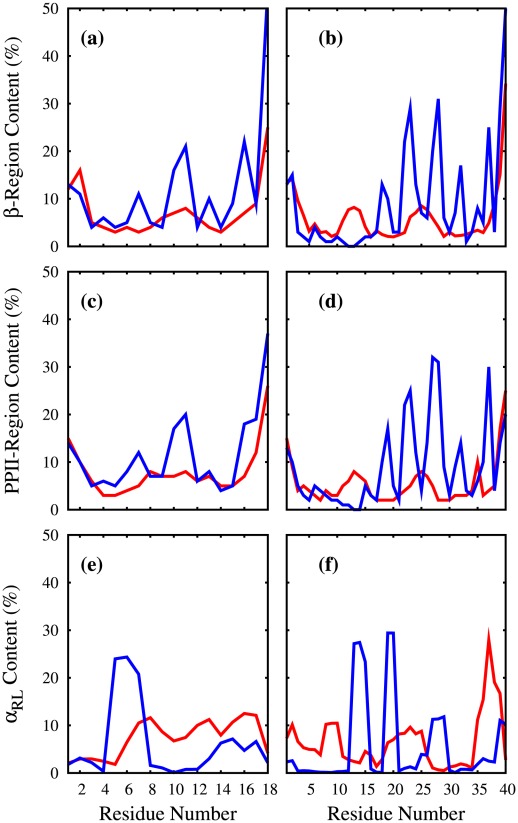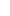, PPII and 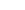 content of selected polyQ peptides. Here, given are the contents (as a percentage) of individual glutamine residues found in: (a,b) 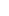-region (c,d) PPII-region (e,f) 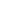. These percentages are plotted against the Glu residue numbers for (a,c,e) 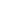 [red], 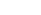 [blue] and (b,d,f) 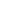 [red], 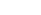 [blue]. These percentages are obtained from clustering the conformations based on their dihedral angles in the Ramachandran plot.

**Figure 3 pcbi-1002501-g003:**
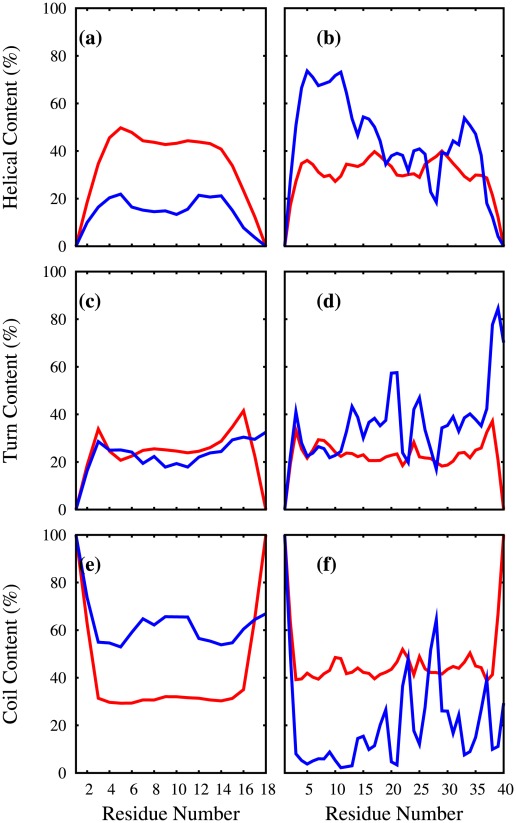
Helical, turn and coil content of selected polyQ peptides. Here, given are the contents (as a percentage) of individual glutamine residues found in the following conformations: (a,b) helical (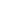,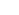) (c,d) turn (H-bonded,bend) (e,f) coil. These percentages are plotted against the Glu residue numbers for (a,c,e) 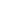 [red],
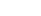
[blue] and (b,d,f) 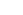 [red], 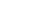 [blue]. These percentages are obtained from the DSSP [58], [59] analysis code.

**Figure 4 pcbi-1002501-g004:**
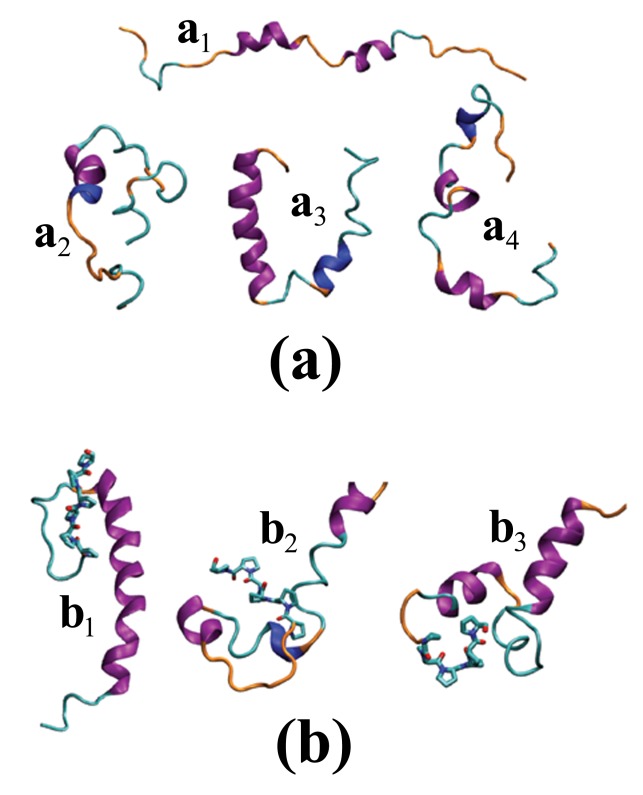
Sample conformations of 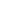
 **and** 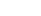 **.** Cartoon representation of sample conformations of (a) 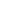 and (b) 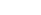. Purple, blue, cyan, and orange represent 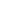-helix, 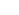-helix, turn, and coil secondary structural motifs, respectively. The licorice-like representation of the proline segment of 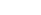 is given in (b). These structures are plotted by VMD [61] using STRIDE [60] for secondary structure prediction.

**Figure 5 pcbi-1002501-g005:**
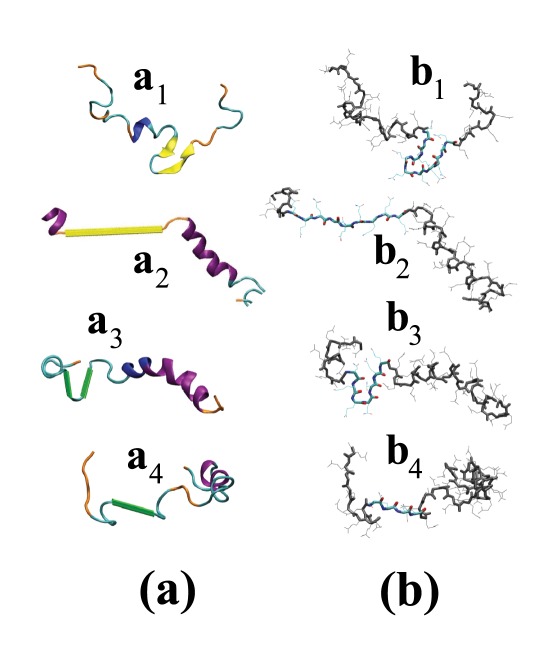
Selected extended conformations of 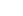
 **peptides.** Here, we give (a) cartoon and (b) licorice-like representation of select conformations of the 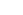 peptide with (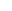,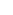,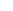,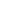) 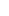 and (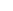,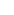,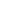,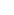) 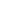 strands. (a) The coloring is similar to Fig. 4 with yellow and green representing 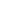 and 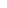 strands respectively. We used a dihedral angle-based algorithm to detect the 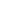 strands and for other secondary structures in these plots we used STRIDE [60] distributed with VMD [61]. (b) The residues involved in (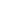) 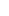-hairpin, (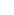) isolated 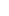-strand, (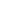) 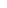-harpin, and (i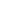) isolated 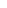-strand are highlighted. The rest of residues are grey and all the side chains are represented by thin lines.

**Figure 6 pcbi-1002501-g006:**
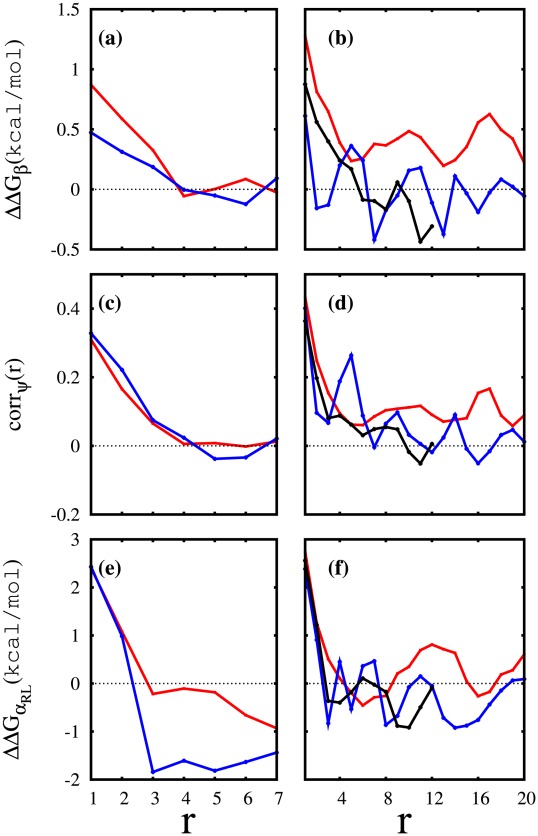
Correlation analysis results for selected polyQ peptides. Here is given the (a) odds ratio based 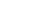 between any two glutamine residues (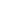 and 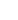) of 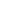 [red] and 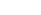 [blue] in terms of (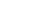). From each side of the peptide 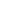 ending residues are omitted in the calculations to reduce the end effects. (b) Similar to (a) for 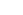 [red], 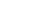 [blue], and 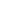 [black]. Here 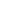 residues from each end are omitted. (c,d) Correlation coefficient between 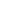 dihedral angles of any two glutamine residues (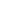 and 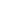) in terms of (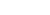) for (c) 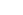 [red], 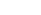 [blue] and (d) 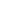 [red], 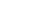 [blue], and 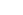 [black]. The end residues were omitted according to the same protocol used for odds ratio analysis. (e,f) Similar to (a,b) but with the odds ratio calculated using the probabilities that residues belong or not to an 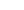 repeat region.

**Figure 7 pcbi-1002501-g007:**
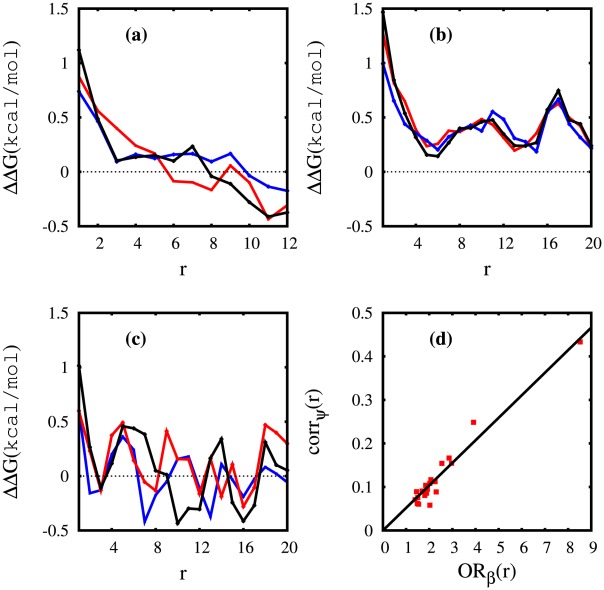
Correlation analysis results for selected polyQ peptides. Specifically, we give 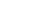 for (a) 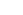 (b) 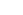 and (c) 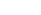 based on OR(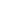)[red] OR(PPII)[blue] and OR(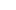)[black]. (d) To compare the linear and OR-based results we plotted 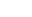
(r) versus the correlation coefficient corr
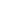
(r) for 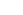 that suggests an almost linear behavior with a correlation coefficient of 0.97.

**Figure 8:**
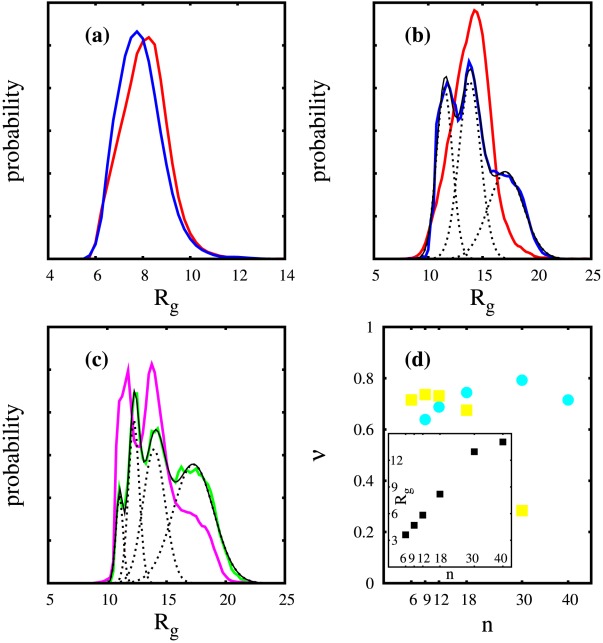
Distribution of radius of gyration of polyQ peptides. (a) The estimated 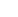 distribution for 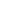 [red] and 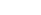 [blue]. (b) The estimated 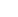 distribution for 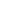 [red] and 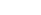 [blue]. The blue curve can be estimated as the sum [black] of three Gaussian distributions [dotted]. (c) The estimated 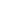 distribution for 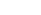, considering only the structures with an all-trans proline segment [green]. Similarly the green curve can be estimated as the sum [black] of four Gaussian distributions [dotted]. Considering only the structures that at least have one cis-proline results in the magenta curve for the 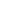 distribution. All the histograms are obtained using a window of width 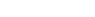. (d) The exponent 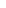 in 
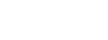
 relation estimated from select pairs of 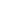 (x axis) and 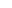 (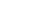 for blue circles and 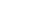 for yellow squares). Inset: The average 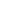 (in 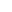) of Q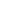 peptides for 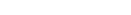.

**Table 2:** Secondary structure analysis of the polyQ peptides.

	(a) Ramachandran regions					(b) secondary structures			(c) extended structures				
peptide	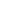	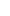	PPII	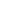	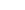	helix	turn	other	PPII	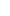 -s	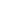 -h	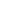 -s	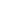 -h
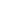	87	5	5	3	7	30	23	47	6.5 (1.3)	7.1 (1.2)	1.1	42 (1.6)	1.9
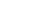	78	9	9	4	6	43	36	21	8.9 (3.3)	3.9 (0.1)	0.5	25 (0.1)	0.1
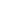	80	8	9	3	7	37	32	31	7.3 (1.3)	4.2 (0.5)	0.5	19 (0.1)	0.7
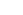	81	7	8	4	7	34	23	43	2.4 (0.3)	1.5 (0.5)	0.5	19 (0.1)	0.2
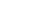	72	13	12	3	6	14	23	63	7.3 (1.0)	0.9 (0.3)	0.1	15 (0.1)	0.1
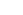	79	8	9	4	8	38	31	31	1.9 (0.2)	0.9 (0.2)	0.3	19 (0.8)	0.1
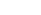	70	14	12	4	6	26	38	36	2.3 (0.3)	1.6 (0.2)	0.1	8 (0.5)	0.0
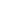	78	9	9	4	8	31	31	38	1.5 (0.2)	0.6 (0.1)	0.4	14 (0.0)	0.0
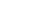	68	15	11	6	10	23	51	26	1.1 (0.1)	1.1 (0.2)	0.6	17 (0.2)	0.0
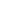	73	12	13	2	4	18	29	53	1.1 (0.2)	1.3 (0.2)	0.0	2 (0.0)	0.0
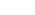	67	17	13	3	8	10	50	40	1.3 (0.2)	1.4 (0.2)	0.0	2 (0.1)	0.0

Here, we give the (a) population (as a percentage) of the residues in the different Ramachandran regions (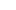, 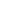, PPII, and 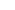), as well as the population of residues involved in 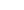 repeats; (b) the population (as a percentage) of residues in different secondary structures (helix, turn, and other secondary structures); (c) the percentage of *conformations* having at least one PPII, 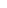, or 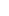 extended secondary structures including isolated strands and hairpins. The isolated 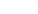, 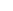, or 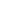 (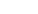, 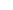, or 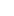) strands – identified in the table as PPII-s, 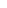-s, 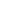-s – are defined based on at least three (four) adjacent residues with the backbone dihedral angles falling into the region associated with these structures; and not involved in any inter-residual hydrogen bonding. Similarly a hairpin – identified in the table as PPII-h, 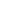-h, 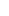-h – is defined based on two adjacent strands of at least three residues with one or more hydrogen bonds between the two strands and a turn in between. For more details of this analysis, that is based on both DSSP [58], [59] and dihedral-based clustering, see *Methods*.

**Table 3:** Helix and turn populations of the polyQ peptides.

	helical content				turn content				
	helix type		helical segments		H-bonding		turn type		
peptide	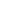	3 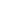	0	1,2,3,4,5	H-bonded	bend	I- 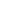	other 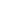	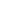
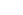	23	7	31	3,16,27,18,4	15	7	18	1	4
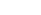	31	12	1	3,21,40,28,6	23	13	24	3	9
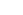	28	9	11	15,37,30,6	22	10	23	2	7
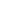	27	7	28	39,31,2	18	6	16	2	5
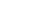	10	4	61	25,13,1	13	10	12	2	9
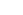	29	9	15	76,9	25	6	25	2	4
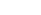	20	6	30	66,4	23	15	28	3	7
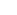	22	9	32	67	25	6	24	1	6
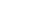	15	8	48	52	31	20	37	4	10
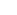	7	11	69	31	24	5	19	2	8
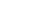	3	7	82	18	25	25	36	4	10

The helical content is partitioned into 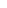
- and 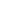-helix populations. The structures are also categorized based on the number of their helical segments. The population of each category (0,1,2,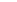) is given if greater than 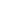
%. The turn content is partitioned based on both the hydrogen-bonding and turn types. For the secondary structure prediction, the DSSP analysis code [58], [59] was used along with the protocols discussed in *Methods*.

### Ramachandran Regions

Figure 1b shows the Ramachandran plot of a typical glutamine residue, for which the clusters in the different regions are computed according to the protocol described in the *Methods* section. Four clusters can be identified in these plots including PPII (blue), 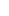 (yellow), 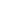 (gray), and 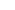 (pink). Figures S2 and S3 show the Ramachandran plots of all 40 glutamine residues of both 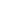 and 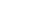. Considering these, as well as similar plots for other peptides (not shown here), we observe the following trends: (i) The dominant region of most residues is the 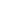 cluster that is present in all residues, except for the glutamines immediately followed by a proline, for which this region is precluded; (ii) PPII and 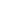 clusters are present in almost all residues; (iii) The 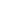 cluster is present in more than half of the residues but its population is often very small; (iv) Compared to 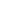, 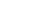 displays regions with higher non-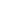 intensities, particularly for the 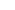 cluster (see 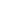, 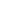, 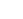, and 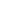).

Figure 2 plots the percent population of the 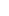, PPII, and 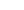 regions of glutamine residues (top, middle and bottom rows, respectively) in terms of the residue number. The left column shows results for 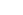 [red] and 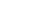 [blue] and the right column for 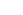 [red] and 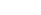 [blue]. Table 2 presents the population of the different Ramachandran regions (averaged over all glutamine residues) and the 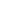 repeats, the secondary structure motifs, and the “extended structures” including hairpins. The residue populations in the Ramachandran plot show that, on average, 67–87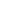 of the residues are in the 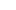 region of the Ramachandran plot, 5–13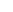 of the residues are in the PPII region and 5–17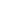 of the residues are in the 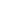 region. The PPII and 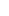 regions are almost always equally probable, as can be seen in Figs. 2, S2, S3. The lowest population belongs to the 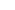 region, comprising only 3–6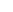 although in certain residues it could be as high as 38% as, for instance, in 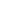 in 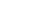 where the content of 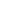 correlates with the presence of turns. The addition of P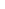 decreases the population of the 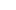 Ramachandran region and increases that of the 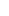 and PPII regions, while leaving the small population of 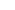 approximately invariant. In 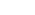 peptides, proline residues are excluded from the statistical analysis so that only Q residue propensities are compared (for instance, when we state that the average helical content of 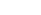 is 43%, it means that 43% of *all Q residues* are in a helix – the P residues are not counted in the statistic).

Figure 2 shows that the populations of the PPII and 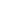 regions are always higher at the two ends of the polyQ peptides, particularly at the C-terminal. When a short proline segment is added at the C-terminal of polyQ, the population of these regions in the neighboring glutamines increases even more. For 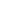 peptides shorter than 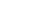 (not shown here), the population of the PPII-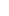 region decreases in the middle of the peptide, but for 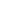 (red line) we see a small peak in the middle of the peptide for both PPII and 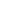 regions. In 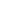, we have two small peaks (rather than a single peak) centered around residues 13 and 25 for both the 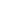 and PPII regions. The presence of the prolines at the C-terminal of a polyglutamine can drastically alter the population distribution. Fig. 2 shows that the few relatively wide peaks of the 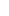-PPII regions in both 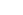 and 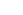 are replaced by several narrow peaks of larger heights. Regarding the residues involved in 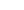 repeats, one can see from Fig. 2e,f that the distribution of these repeats throughout these peptides depends both on the position of glutamine residues and the presence or absence of the C-terminal prolines although, as seen in Table 2, the average 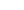 content is similar (6–7%) in all four peptides: 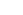, 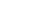, 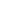, and 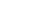. We note that the distribution of 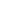 content in the peptide is mostly determined by the 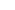 content as the 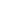 content is abundant in these peptides and most 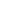 residues are involved in an 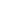 repeat. One can compare Fig. 2e,f with Figs. S2,S3 and observe similar behaviour, *i.e.*, the residues with high 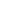 content (Fig. 2e,f) have more intense 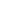 clusters (pink clusters in Figs. S2,S3).

### Secondary Structure

When one considers not only the backbone dihedral angles *i.e.*, the (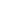,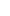) regions occupied by individual glutamine residues, but also the inter-residual hydrogen bonding and the relative positions of the 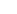 atoms, one can identify different secondary structures, particularly 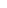-helical segments in many of the sampled conformations. Short 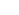 helices are also possible but the majority of the residues are either in a turn or a coil conformations according to both DSSP [58], [59] and STRIDE [60] analysis. Figure 3 plots the helical, turn, and coil content of the individual glutamine residues against their residue numbers for 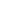, 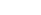, 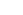, and 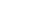. Figure 4 shows plots of select conformations of 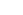 and 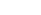 peptides, as generated by VMD [61] using STRIDE [60] for the secondary structure assignment. Table 2 lists the population of helix, turn, and “other” secondary structures as obtained from DSSP, averaged over all residues. The “other” secondary structure category includes mainly what DSSP identifies as “loop or irregular” – sometimes called “coil” in other programs – but which may also include a very small population of other secondary structures such as extended 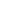 strand and “isolated 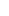-bridge”. We use the protocols explained in *Methods* section to further identify these, as well as other extended structures (Tables 2 and 3).

When the population of residues in the 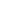 region is compared to the actual helical content, one realizes that the majority of the residues in the 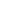 region do not form 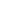 or any other type of helices. Many of these residues in the 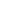 region are followed and/or preceded by a residue in a different Ramachandran region, such as 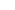, as discussed in the previous subsection, forming an 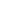 repeat. Similarly an 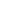 repeat does not necessarily form an 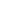 strand. Table 2 gives the population of the structures (or conformations) having at least one segment in one of the extended conformation forms, as defined in *Methods* section, including 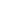 and 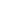 strands either in the isolated form of length 3 (or length 4 in parenthesis) or in the hairpin form as well as PPII structures of length 3 (or length 4 in parenthesis). Note that unlike the other populations in part (a) and (b) in Table 2, the population of extended secondary structures in part (c) is not averaged over the residues. Instead, we counted all the conformations having *at least one* such secondary structures in the polyQ portion of the molecule and divided this number by the total number of sampled conformations. These structures are less common than helices or turns, but they are possible and form a small subpopulation of the secondary structures. Indeed, one can see that a non-negligible portion of the structures has at least one such segment. In particular, isolated 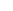 strands are quite common, although they may simply be considered as part of a random coil. The isolated 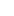 and PPII strands form the second most populated extended structures. Similarly, these structures may also be considered as part of a random coil. However 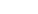, 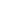 and 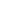 strands form extended structures that are unlikely to be considered random coil elements. Figure 4 shows some examples of isolated and adjacent extended structures in both 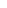 and 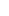 forms.

Remarkably, among all the sequences presented here, 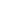 has the highest percentage of extended structures. This peptide shows a significantly higher propensities for the extended structures, particularly the 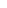 strands. The population of the structures having at least one 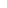-hairpin is almost 2%, and is higher than the number of structures having at least one 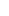-hairpin. However, the 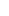-hairpin rate is still the highest among all the peptides studied here. Adding the proline segment to the 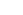 peptide reduces the chance of forming 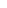 or 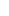 extended structure dramatically, especially in the case of 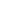-hairpins and isolated strands of length four or more. However, PPII propensity is increased in the peptides of length 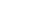 by adding the proline segment.

Table 3 gives more details on the helices and turns observed in the polyQ and polyQ-polyP structures. The helices are found mostly in the right-handed 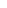 form except for 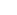 and 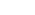 that favor 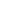 helices due to their short length. This Table also shows the percentage of helical segments present in a given peptide. A helical segment is defined as a series of residues adjacent in the sequence whose secondary structure has been identified as helical by DSSP. Thus helical segments can have varying lengths, and the table lists the number of helical segments (independent of their length). Thus, among 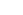 conformations, 31% do not have any helical segment but when the prolines are added 99% form at least one helical segment (in particular, 40% of the structures in 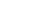 have 3 helical segments). The addition of P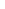 to 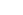 increases the helical content from 30% in 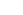 to 43% in 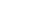 (the highest helical content in all peptides), while the addition of polyP decreases the helical content in all other peptides. Comparing 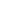 and 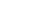 structures, the population of the structures having more than one helix increases.

The select 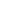 and 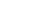 structures given in Fig. 4a,b illustrate various conformations, for which a statistical description is given in Figs. 2,3 and the Tables 2–3. In particular, the left column of Fig. 3 indicates that adding a polyP segment to 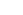 reduces the helical content but increases the coil content (while the turn content stays the same). Instead, adding a polyP to 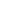 (right column of Fig. 3) results in an increase of the helical content in the N-terminal of 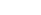, farther away from the polyP segment. The addition of 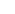 to 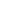 increases not only the number of helical segments but also their length, particularly in the N-terminal half. The population of the structures having short helices (less than 7 residues) is very similar in 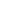 (26%) and 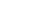 (27%) but 72% of 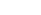 conformations have longer helices (7 residues or more) as compared to only 43% in 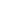. Also 37% of the 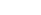 conformations have a helical segment longer than 9 residues while only 20% of 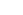 conformations do.

Adding the polyP segment generally increases the turn content (both of 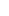 and 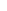 types), except for 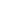, where the total population of turns stays constant. The majority of turns are of I-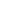 type but there is a smaller population of other types of 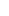 turns as well as 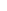 turns. The increase in the 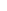-turn content of polyQ-polyP peptides can explain why adding the polyP to polyQ sometimes increases the 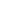 content, as 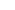 residues are involved in most of 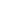-turns. For instance, one finds more 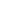 content in the residues of 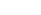 compared to 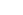 but there are fewer residues in 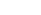 involved in 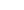 repeats. There is no contradiction here as part of the 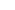 content is involved in 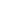 turns rather than 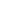-strands. Finally, Fig. 5 presents examples of (rare) extended conformations in the 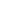 peptides. In particular, the figure shows 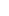 hairpins and isolated strands, and 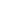 hairpins and isolated strands.

### Correlation Analysis

An odds ratio analysis based on the Ramachandran regions was conducted, and results summarized in Figures 6 and 7 for 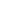, 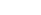, 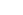, 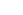, and 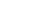 peptides. We defined the OR as a function of sequence distance 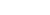 between two glutamine residues 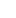 and 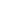. 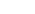 indicates an OR based on the 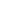 region of Ramachandran plot. These figures display 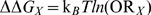, for a better intuitive illustration. 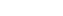 measures how the presence or absence of 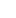 in the 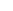 region can influence the presence or absence of 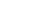 in the 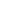 region. Here, to reduce the end effects, 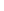 only runs between 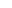 and 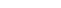, with 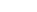 for 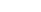 and 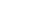 for 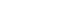.

In Fig. 6a, 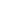 shows higher correlation 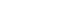 for 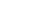 than 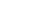. In other words, 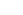 would have a greater chance of forming 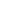 strands if the population of 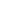 residues increases. However the correlation range between the 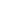 residues in both 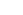 and 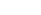 is about 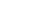 since for 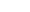 there is no significant deviation from 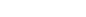, the expected value for independent events. This situation changes with polymer length. 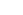 in Fig. 6b has a correlation length of about 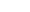, after which it quickly loses correlation (it even becomes “anti-correlated”). Once again, 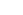 exhibits unique behavior since 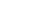 does not decay to zero but oscillates around 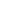 kcal/mol and more importantly, the oscillation does not seem to be damped by increasing 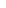 (ignoring the smaller 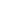 values). This indicates a long-range correlation between the glutamine residues of 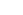. (Oscillations can be seen for 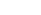 as well, but they are around zero).

The results of the OR analysis can be further confirmed by conducting a direct correlation analysis on the 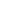 angles of the glutamine residues. We used the correlation coefficient (also known as cross-correlation or Pearson correlation) as a measure of linear correlation between the 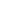 angles of Gln residues of sequence distance 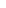, using the same protocol explained above for odds ratio analysis (*i.e.*, omitting the end residues) and verified the same unique behavior of 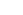. First, the 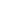 dihedral angles were shifted 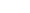 degrees (with the assumption of periodic boundary condition at 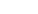), then the correlation coefficient of 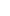 of the residues with a sequence distance 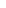, corr
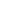
(r), was calculated. Note that this correlation measure does not involve any clustering and ignores any dependence on the 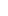 dihedral angle, however, it confirms the OR predictions. Although in general both 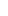 and 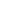 angles are needed to identify the Ramachandran region of an amino acid, the linear correlation analysis on 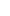 angles is still able to detect a long-range, positive correlation for 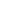 (Figs. 6c,d).

An OR-based correlation analysis for 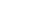 is illustrated in Fig. 6e,f. Here, a residue is considered to be an 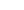 residue if it is involved in an 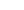 repeat. In the case of 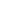 and 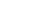 there is an even shorter positive correlation range (compared to 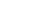) for both peptides, with a significant negative correlation when 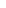 increases. 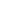 shows a somewhat similar oscillatory behavior around a non-zero average, with negative troughs. Note that the Pearson correlation coefficient cannot be used here for the 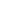 analysis (in its univariate form) due to the fact that the definition of an 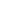 repeat is highly dependent on the dihedral angles of both adjacent residues, involving four residues in the correlation analysis instead of two. The 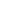 angles are also quite important for the 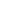/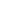 distinction.

Finally, Fig. 7 compares the behavior of OR-based 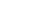 in 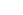, 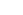, and 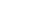 peptides for 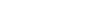. In 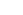 there are differences between these different regions, but they all decay by increasing 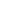, as expected for short correlations. However, in 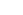 we see an almost identical behaviour for all three Ramachandran regions. This clearly indicates that the dihedral angles of most of the glutamine residues are correlated in an indirect manner, influencing each other. We compared the 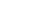 of glutamine residues based on their distance 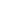 and the correlation coefficients of their 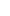 angles for 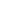. Fig. 7d shows that the two vary similarly for different 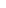 and have a correlation coefficient of about 0.97, suggesting that OR and corr are linearly correlated.

In terms of the error estimate, we note that the estimated standard error for these calculations is different not only for different plots but also for different data points (varying by 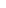) in one plot. The latter is the result of having fewer samples with larger 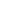 than shorter 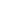 but the former is due to the difference between the population of secondary structures, the number of residues in each peptide, and the number of sampled conformations for each peptide. However, the standard error remains less than 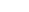 kcal/mol in most cases. In some exceptions in Fig. 6e,f the standard error could be as high as 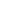 kcal/mol.

### Radius of Gyration

Here we consider the statistical ensemble results concerning the radius of gyration and its distribution. The radius of gyration 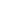 gives a simple and intuitive measure of the overall structure of the polyQ peptides as the collapsed (stretched) structures are associated with smaller (larger) values of 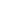. Table S1 gives the 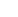 of the 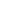 atoms of the Gln residues in 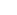 and 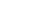. The proline segments are not included in the calculation of 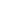 so that the polyQ sequences are compared on equal footing. The averages are accompanied by the standard deviation that somewhat estimates the width of the distribution, if it is close to a normal distribution. The averages do not show much difference between 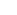 and 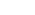 peptides. The standard deviation is also very similar between the two in most cases except for the case 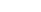. Fig. 8a shows the 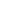 distribution of 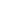 [red] and 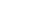 [blue] peptides that is close to a normal distribution with a longer tail on the right as expected for a random-coil structure. 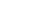 is only slightly more compact. The normal distribution with a slightly longer tail as a characteristic distribution of random coil is seen for all of these peptides except for 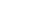. Fig. 8b shows that although 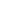 follows the same distribution, 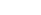 can be estimated as the sum of three distinct Gaussian distributions.

We used the Marquardt-Levenberg [62] algorithm to estimate the probability distribution of 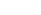 as the sum of three Gaussian distributions (see Fig. 8b), each representing one class of structures covering 24, 44, and 32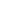 of the samples distributed around an 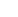 of 11.41, 13.65, and 17.08 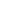, respectively. The fitting resulted in a reduced 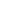 smaller than 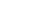, indicating that this model explains the probability distribution of 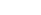 well. Examining the structures of each class shows that the 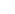 segment is responsible for this clear difference between the three classes. The structures distributed around 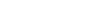, accounting for almost one third of the samples, have relatively stretched conformations (see Fig. 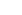), and this correlates with the presence of all-trans prolyl bonds in 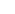. In these proline isomers, 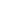 forms a rigid stretched helical segment, in contrast with a proline segment including one or more cis-isomers, particularly in the middle of the segment (see Fig. 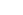). Table S1 shows the trans content of each of the prolyl bonds of 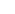 as well as the population of the 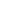 isomers with all-trans prolyl bonds. There is a clear difference between 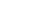 and the rest of proline-containing peptides in terms of cis-trans isomerization. Although, 73–77% of the residues are in trans conformation in the shorter peptides, only 12–23% of the structures are all-trans. In 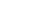 60% of the structures are stretched all-trans conformations. What is more interesting is that the distribution of radius of gyration is meaningfully different for the all-trans proline sub-ensemble as shown in Fig. 4c. Green curve is the 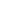 distribution of this sub-ensemble and magenta curve is the 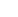 distribution, obtained from the rest of the structures (*i.e.*, cis-containing polyP). Here we somewhat recognize four normal distributions. We use a similar method as explained above to fit these Gaussians. We find four clusters with 6, 17, 29, and 48% of the population centered around 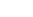
11.02, 12.24, 13.94, and 17.27 respectively. The conclusion is that all-trans prolines increase the population of the stretched cluster considerably. This somewhat explains why we do not observe this partitioning of the clusters with proline segment in shorter peptides (see Fig. 8a) because in those cases the population of all-trans conformations is not large enough to affect the overall 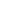 distribution.

As the peptides 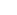 grow with residue number 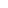, their structure becomes more collapsed. In particular, the average radius of gyration for 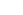 is only about 1.1 Å larger than for 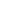. The inset in Fig. 8d illustrates the dependence of the radius of gyration on the length of the peptide. Assuming 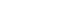 one can estimate 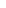 using any pair of peptides such as 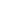 and 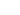 from 
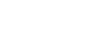
. Fig. 8d gives examples of the estimated 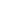 for different pairs of 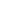 and 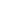: 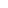 is given by the indices in the x axis and 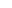 is 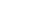 (cyan circles) or 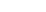 (yellow squares). There is an abrupt collapse of the structure (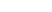) on going from 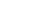 to 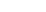.

## Discussion

Our atomistic simulations show the disordered nature of monomeric polyglutamine peptides, in agreement with experimental conclusions [6], [13]–[15] and with previous all-atom MD simulations [35]–[38]. Our simulations are also in agreement with recent experiments [18] in that the monomeric polyQ is different from a total random coil or a protein denatured state, with a significant presence of short 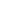-helices. Therefore polyglutamine is a disordered peptide that is somewhat preorganized, containing short rigid segments [63], [64]. Contrary to certain coarse-grained models [27]–[29], [31], our atomistic simulations provide no evidence for a large 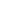 content in monomeric polyglutamines.

We observed that the 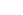 peptide forms an ensemble of mostly compact structures with an average radius of gyration only about 1.1 Å larger than that of 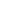. This agrees with the conclusions from single-molecule force-clamp experiments [24] that polyQ chains collapse to form a heterogeneous ensemble of globular conformations that are mechanically stable. For the radius of gyration of the shorter peptides, we observed an exponent 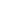 slightly larger than that of a random-coil in a good solvent (*i.e.* about 0.6, [65]). However, we have not been able to simulate a large enough range of peptide sizes in order to get a good estimate of 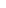. This may not be necessary, since the simulations suggest that the radius of gyration does not follow a power law anyway (see Fig. 8d).

The addition of a short C-terminal proline segment to the 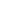 peptide changes the distribution of the radius of gyration from a Gaussian-like function with a longer tail for larger 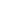 – a characteristic of a random coil, seen also in all the other peptides studied here – to a combination of three distinct Gaussians. The way the proline segment affects the 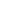 distribution is closely correlated with the cis-trans pattern of its prolyl bonds. An all-trans proline segment (the most common pattern in 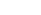) results in the multi-modal distribution of Fig. 8. Instead, proline isomers with cis bonds are abundant in shorter peptides which results in the normal 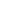 distribution. We note that prolyl bond isomerization requires crossing barriers of 10–20 kcal/mol, which can only be accomplished with special enhanced-sampling techniques such as used here [44], [47], [49].

The addition of the polyP segment to polyQ introduces position dependent features among the Gln residues. This is readily seen in Fig. 3. The fluctuations observed cannot be explained as “noise” resulting from sampling limitations. As explained in the previous section, sampling of independent data produces the same features, which suggests a sensitive dependence on the position of the residue in the sequence. Interestingly, polyP induces helix formation in the further residues in the N-terminal of 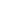, while creating more turns in the nearer Gln residues. As a result of the polyP addition, the overall 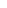-helical content of 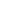 increases. This is in contrast with the shorter peptides in which the 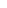-helical content drops considerably by adding the polyP segment.

Experimentally, it has been claimed that the addition of polyP to polyQ decreases the 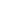-helical content of polyQ for all polyQ lengths [17]. A superficial comparison might indicate that this is in contradiction with our results for 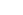. Our results are, however, in agreement with the experimental data, which is based on the CD spectra of these peptides. These CD spectra identify the distribution of individual backbone dihedral angles rather than the actual 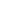-helical content, a quantity not only dependent on the individual residues but also the way they are aligned. Our simulations are in total agreement with this observation as we see a decrease in the population of the 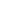 cluster (*i.e.*, the residues falling into the 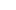 region of Ramachandran plot) in all the peptides studied here, as we add a 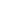 segment to the C-terminal (Table 2). As we have pointed out before [41], [42], care is needed in the interpretation of the CD data. Table 2 shows that the majority of the residues in the 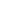 cluster are not involved in any form of helix in either polyQ or polyQ-polyP peptides, and while the helical content of all other peptides decreases, that of 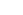 actually increases with the addition of 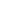. While this effect for 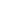 cannot be ruled out as an defficiency of the force field, it is interesting to note that this would represent quite an effective way of neutralizing 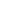, since the rather stable 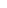 helix will not be prone to aggregation.

In addition to 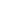 and 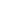 helices, as well as 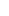 and 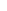 turns, one can identify a small but non-negligible population of extended secondary structures of 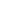 and 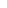 strands, particularly in the 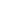 peptides. PolyP increases the 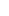-region content in the Ramachandran plot, but decreases the 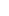-strand content (as explained before, several 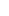 residues need to be adjacent in order to form a 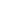-strand). For 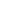, the addition of polyP dramatically decreases the content of 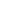, 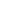, 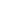 and 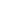 strands. On the other hand, relatively short PPII helices in polyQ form another extended secondary structure that happens to be more common in 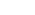 peptides than 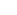 peptides for 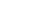. The PPII strands do not form inter-residual hydrogen bonds (hairpins,sheets) and would not favor aggregation.

In this work we used an odds ratio analysis to quantify the dependencies among certain properties of the molecules. Regarding the 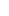-strand formation in 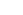, the graph for 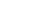 in Fig. 6 shows a positive, long-range correlation in sequence distance. In other words, the chances of two glutamine residues falling into the 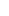 region of the Ramachandran map correlate positively with each other, even if they are distant in the sequence. This long range correlation was not seen in any other peptide but 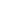. Interestingly, this long-range correlation for the 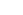 peptide is not limited to the 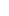-region but it is also seen in other regions such as 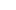 and PPII. In particular, 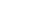 *scales* for the 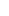, 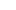 and PPII regions as shown in Fig. 7. A linear correlation analysis on 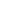 dihedral angle verifies the very same long-range correlation between glutamine residues of 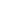 peptide, a correlation that is absent in other peptides studied here. This surprising phenomenon could be interpreted as the possibility of the growth of any of these secondary structures in the long polyQ peptides, especially if the conformation were “seeded” with a given secondary structure. In a polymeric form of polyglutamine, the nucleation of 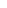 or 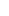 strands could result in further growth of those strands or could induce growth in adjacent strands resulting in the the growth of 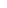 or 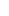 sheets. Interestingly, the “period” for the oscillations of 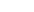 is approximately 7–8 residues, which is also the optimal experimental extended chain length in an aggregate [7].

The populations of 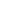-strand, 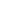-strand, 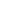-hairpin, and 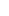-hairpin (Table 2) decrease and the long-range correlations 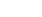 and 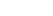 are disrupted by the presence of the C-terminal proline residues in 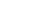. For shorter peptides, the corresponding populations are much lower, and the 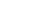 correlations are short-ranged. Taken together, these results indicate that for 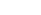 (but not for the shorter peptides) nucleation could start in one of these strands or hairpins (that can align two strands) and then grow from there, favored by the positive correlations generated by the longer peptide.

We can summarize the main findings of this work as follows:

1. *Monomeric* 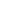 *peptide forms an ensemble of disordered, mostly compact structures with non-negligible* 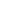 *helical content and other secondary structures, and with a very slow growth of the radius of gyration with the number of peptides for longer polyQ peptides*. This is in agreement with previous experimental and simulation results [6], [13]–[15], [24], [35]–[38]. The average radius of gyration of 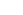 is only about 1.1 Å larger than that of 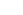.
2. *The average radius of gyration for polyQ does not vary with the addition of polyP, but its distribution in* 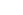 *is affected by the isomerization states of the polyP segment*.
3. *For peptides of all lengths, the population of the* 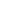 *region in the Ramachandran plot decreases while the populations of the* 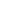 *and PPII Ramachandran regions increase with the addition of polyP*.
4. With respect to secondary structures (i.e., dihedrals angles and hydrogen bonds, *the addition of polyP increases the PPII and turn contents, and decreases the helical content in all peptides but* 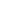. These effects probably disfavor aggregation as PPII structures dislike backbone H-bonding, turns increase disorder, and the increase of helical content in 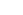 may also disfavor aggregation as helices are quite stable, with all their H-bonds properly engaged.
5. *Although small, the populations of* 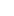 *,* 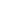 *,* 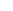 *and* 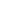 *strands, as well as* 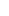 *-hairpins and* 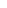 *-hairpins, are considerably larger for* 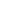 *than for smaller peptides*. These populations decrease when polyP is added. These small secondary structures are good candidates to initiate nucleation: the strands might “attract” other strands to hydrogen bond and the hairpins help to align two strands. Their suppression by the presence of polyP would disfavor aggregation.
6. An odds-ratio based correlation function 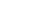 describes how the chances of two Gln residues of falling into a given region of the Ramachandran plot correlate. *Only* 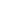 *shows positive*, **long-range** *correlation in sequence space for various regions of the Ramachandran plot. The addition of polyP destroys this long-range correlation for* 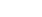 *and* 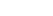 *.* In particular, 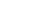 *scales* for the 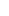, 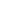 and PPII regions. Together with the results described in (6) above, this could be interpreted as the possibility of the growth of the 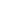 or 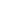 strands or hairpins already present in disordered 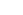 (or longer polyQ peptides). Interestingly, the “period” for the oscillations of 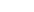 is approximately 7–8 residues, which is also the optimal experimental extended chain length in an aggregate [7]. A linear correlation analysis on 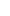 dihedral angles confirms this period is a “universal” feature of correlations in long polyQ peptides.

Our careful statistical analysis has revealed a wealth of very subtle effects that are far from obvious. Secondary structures such as 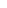 helices, 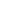-sheets, 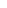-sheets, PPII helices, and coils have all been reported in the literature. The picture that is emerging is that if one can induce the nucleation of one of these structures, or provide a template for it, a long enough polyQ polymer or an aggregate will probably continue growing in the given conformation, even if it is not the absolute thermodynamic minimum. In this sense, the wealth of conformations of polyQ is reminiscent of the different phases that appear in ‘inorganic’ systems with short-range attractive interactions and long-range electrostatics interactions such as Langmuir monolayers or block copolymers, where *kinetics* effects also play a fundamental role in determining the final phase of the system. PolyQ is a very special homopeptide due to its long side changes and the dipoles at the ends. The van der Waals packing of the side chains provides the source of short-range attractive interactions, while the carboxamide groups provide the long-range dipolar interactions [34]. In this sense, the only other peptide that would exhibit similar behavior is asparagine, with one methyl group less in its side chain [34]. The “collapsed” random coil would just represent the *frustration* between different phases.

## Supporting Information

Figure S1

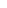
-helical content of 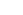 and 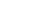 peptides. Here, we give (a,b) the 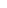-helical content (as a percentage) of individual glutamine residues plotted against their residue numbers for 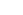 [red] and 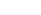 [blue] as obtained from the last 100 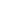 of two 200 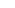 long independent simulations; (c,d) The 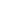-helical content (as a percentage) of individual glutamine residues plotted against their residue numbers for 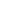 [red] and 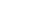 [blue] as obtained from the third (c) and the fourth (d) 250 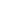 of 1000 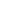 REMD simulations.(EPS)

Figure S2Ramachandran plots of Gln residues in the 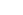 peptide. On these plots, each pixel represents a 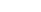 bin, whose intensity represents its relative population, ranging from 1,2,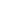, 49, and 50 or more samples out of 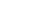 conformations. Color scheme is as in Fig. 1.(EPS)

Figure S3Ramachandran plots of Gln residues in the 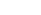 peptide. See Figures 1 and S2 for the details.(EPS)

Table S1 Radius of gyration and cis-trans isomerization.
(PDF)

Text S1 This text includes a description of our simulation details, secondary structure assignments, and radius of gyration analysis.
(PDF)
