# Supplementary material for: Are Long-Range Structural Correlations Behind the Aggregration Phenomena of Polyglutamine Diseases?
Source: PLoS Comput Biol. 2012 Apr 26;8(4):e1002501. doi: 10.1371/journal.pcbi.1002501 (PMC3343152; doi:10.1371/journal.pcbi.1002501)
Supplement: Table S1 — Radius of gyration and cis-trans isomerization. (PDF) [file pcbi.1002501.s004.pdf]

Table Table S1. Radius of gyration and cis-trans isomerization.

	$R_g(Q_n)$		$R_g(Q_nP_6)$		trans content							all-trans
n	mean	st.dev.	mean	st.dev.	P^1	P^2	P^3	P^4	P^5	P^6	**average**	population
40	14.06	1.80	14.45	2.41	85	88	98	92	93	96	**92**	60
30	12.96	1.71	-	-	-	-	-	-	-	-	-	-
18	8.20	0.96	8.01	0.95	68	67	69	80	87	91	**77**	23
12	5.83	0.65	5.81	0.67	69	68	68	78	87	94	**77**	22
9	4.69	0.57	4.47	0.53	71	64	63	82	89	93	**77**	18
6	3.62	0.57	3.73	0.67	73	73	52	64	84	91	**73**	12

The mean radius of gyration and its standard deviation (in $\AA$) for Q_n and Q_nP_6 peptides, along with the trans content (as a percentage) of all prolyl bonds of Q_nP_6 peptides along with their averages are given. The population of all-trans structures for each Q_nP_6 peptide is given as well.
