# Supplementary material for: Are Long-Range Structural Correlations Behind the Aggregration Phenomena of Polyglutamine Diseases?
Source: PLoS Comput Biol. 2012 Apr 26;8(4):e1002501. doi: 10.1371/journal.pcbi.1002501 (PMC3343152; doi:10.1371/journal.pcbi.1002501)
Supplement: Text S1 — This text includes a description of our simulation details, secondary structure assignments, and radius of gyration analysis. (PDF) [file pcbi.1002501.s005.pdf]

Are long-range structural correlations behind the aggregration phenomena of polyglutamine diseases? (Supporting Text)

Mahmoud Moradi[1], Volodymyr Babin[1], Christopher Roland[1], Celeste Sagui[1,*]

1 Center for High Performance Simulations (CHiPS) and Department of Physics, North Carolina State University, Raleigh, NC 27695-8202.

$*$ E-mail: sagui@ncsu.edu

Simulation Details

Simulations were carried out for the peptides of sequence $\mathrm{Ace}-(\mathrm{Gln})_n-\mathrm{NH_2}$ (denoted as Q_n) and $\mathrm{Ace}-(\mathrm{Gln})_n-(\mathrm{Pro})_6-\mathrm{NH_2}$ (denoted as $\mathrm{Q}_n\mathrm{P}_6$). These peptides include Q_{40}, $Q_{40}P_6$, Q_{30}, Q_{18}, $Q_{18}P_6$, Q_{12}, $Q_{12}P_6$, Q_9, Q_9P_6, Q_6, and Q_6P_6. In each case, we refer to the ith glutamine and jth proline residues as Q^i and P^j, respectively.

Initial configurations consisted of the unfolded peptides, which were generated using the LEAP program of the AMBER v.9 simulation package. The simulations used the ff99SB version of the Cornell *et al* force field [1]. The leap-frog algorithm with a 1 fs timestep was used along with the Langevin dynamics. All the simulations were carried using an implicit water model based on the Generalized Born approximation (GB) [2, 3] including the surface area contributions computed using the LCPO model [4] (GB/SA). For this model, a cutoff of 18 Å was used for the nonbonded interactions. For GB, it has been shown [5] that such a large cutoff generates results compatible to those obtained with no cutoff. Our use of an implicit solvent model is due not only because of the very large computational costs associated with simulating an explicit solvent environment, but also because our simulations are based on a replica exchange sampling scheme. Our particular replica exchange scheme uses temperatures as high as 1200K, which is clearly not compatible with any explicit solvent model. Since the use of such a replica exchange scheme turns out to be crucial in terms of being able to adequate sample equilibrium configurations (especially those involving rare structures), we opted for an implicit solvent model.

For polyQ peptides we used the T-REMD scheme using 20 replicas for $n = 6, 9, 12, 18$ and 24 replicas for $n = 30, 40$. The temperatures were distributed as: 300, 322, 347, 373, 401, 432, 464, 499, 537, 578, 622, 669, 720, 774, 833, 896, 964, 1037, 1115, 1200 K for $n = 6, 9, 12$ and as: 300, 319, 340, 362, 386, 411, 438, 467, 498, 530, 565, 602, 641, 683, 728, 776, 826, 880, 938, 1000 K for $n = 18$ and as: 300, 316, 333, 351, 369, 389, 410, 432, 456, 480, 506, 533, 562, 592, 624, 657, 693, 730, 769, 811, 854, 900, 948, 1000 K

for $n = 30$ and as: 300, 313, 326, 340, 355, 371, 387, 404, 421, 440, 459, 479, 500, 522, 545, 568, 593, 619, 646, 674, 703, 734, 766, 800 K for $n = 40$.

For polyQ-polyP peptides we used the HT-REMD scheme. Therefore we first ran ABMD simulations to generate biasing potentials. The initial biasing potentials were transferred from our previous ABMD simulations of short polyproline peptides [6]. We refined these biasing potentials for each Q_nP_6 peptide separately by running ABMD simulations with the same number of replicas and the same temperature distribution as for the T-REMD simulations of the corresponding Q_n peptide. The refined *one-dimensional* free energy maps formed the basis of the HT-REMD runs for enhanced equilibrium sampling. Four more replicas were then added, all at $T = 300$ K: one with no biasing potential, and three with the ABMD generated biasing potential scaled down by a factor of 0.49, 0.76 and 0.9, respectively. The choice of temperatures, the scaling factors, and the ratio of temperature-varying versus Hamiltonian-varying replicas was to ensure a similar rate of exchange between all neighboring replicas.

We ran 200, 400, and 1000 ns REMD simulations for the Q_n and Q_nP_6 peptides of $n = 6, 9, 12$, $n = 18, 30$, and $n = 40$, respectively. Coordinates of the unbiased $T = 300$ K replica were sampled every picosecond. In all the cases except for $n = 18$, only the second half of the sampled structures were used for the analysis to ensure that the ensemble is not dependent on the initial structures. For $n = 18$, we ran two completely independent 100 ns long simulations for each peptide (Q_{18} and $Q_{18}P_6$) and combined the second halves of the two independent simulations for the analysis.

Finally, we discuss the convergence of the simulations. How can we be sure that the conformations obtained in our simulations are sampled correctly? Computational limitations preclude us from running longer, which can be important for the longer peptides with long folding times. First, we need to make sure that the REMD scheme, used for both Q_n and Q_nP_6 peptides, resulted in a reasonable rate of exchange between the neighboring replicas. This rate varied as 50-55, 41-55, 38-46, 33-41, 31-42, 31-41, 30-44, 28-43, 26-37, 25-37, and 26-35 % for Q_6, Q_6P_6, Q_9, Q_9P_6, Q_{12}, $Q_{12}P_6$, Q_{18}, $Q_{18}P_6$, Q_{30}, Q_{40}, and $Q_{40}P_6$, respectively, confirming a reasonable performance. We can also check the convergence of the statistical measurements based on the following idea. We split the data used for the analysis in two (still large) parts. We ran the analysis on the two parts separately and compared the numbers/plots obtained from them. The data thus obtained was consistent with the data presented here. In the case of $n = 18$ we even ran two completely independent simulations as further check.

Figure S1a,b shows the α-helical content (as a percentage) of individual glutamine residues plotted

against their residue numbers for Q_{18}[red] and $Q_{18}P_6$[blue] as obtained from the last 100 ns of two 200 ns long independent simulations. The two plots are qualitatively similar although there are some insignificant differences between them. Note that since our main goal was to better understand the molecular origin of aggregation and its suppression, we concentrated more on the case of $n = 40$. Our data for $n = 40$ shows a good convergence. Fig. S1c,d shows the α-helical content (as a percentage) of individual glutamine residues plotted against their residue numbers for Q_{40}[red] and $Q_{40}P_6$[blue] as obtained from (c) the third and (d) the fourth 250 ns of 1000 ns REMD simulations. Not only the overall behaviour is similar, but also the variations with the residue number are consistent, and would indicate a sensitive dependence on the position of the residues in the sequence.

Secondary Structure

We used the (ϕ, ψ) dihedral angles (see Fig.1 for their definition) to identify different regions [7] of the Ramachandran map [8]. According to this scheme, the α region is divided into two parts: α_R defined by $-120° < \psi < 90°$ and $-160° < \phi < -20°$ and α_L defined by $-50° < \psi < 110°$ and $20° < \phi < 160°$. PPII region includes $-110° < \phi < -20°$ and ($90° < \psi < 180°$ or $-180° < \psi < -120°$). The β region consists of two parts: ($-180° < \phi < -110°$, $90° < \psi < 180°$ or $-180° < \psi < -120°$) and ($160° < \phi < 180°$ and $120° < \psi < 180°$) regions.

Although this delineates clear regions for the dihedrals of most residues, it turns out that there is considerable overlap between the populations of the PPII and β regions. In some cases the α_R region may overlap with both the PPII and β regions as well. In order to handle this situation, we used a clustering technique to identify the region of the conformations, rather than strictly enforcing the sharp boundaries of the defined regions. These two methods give identical answers for most dihedrals. However, at the borders, the clustering technique is more accurate. We used a central clustering method, also known as vector quantization [9] technique, with the stochastic implementation of the Expectation Maximization (EM) [10] method in an algorithm that is reminiscent of the widely used K-means algorithm [11]. This is a non-parametric data clustering technique that employs the iterative EM algorithm in a stochastic manner. We initially used the regions, defined above, to identify the secondary structure of each sampled conformation. We defined four clusters: PPII, β, α_R, and α_L. Next, we defined an association function, z_c^i as the probability that the conformation i belongs to the cluster c. Initially, these functions are either 0 or 1 based on which region the conformation occupies. In an iterative manner, we optimized the association

functions for each conformation, using a Gibbs measure $z_c^i \propto \exp(-s(D_c^i)^2)$, with s representing a softness parameter and D_c^i the distance of (ϕ,ψ) of the ith conformation to the reference point of the cluster c, defined as the average of all the (ϕ,ψ) dihedrals of the conformations weighted by z_c^i. Note that the distance here is defined under the periodic boundary condition. The parameter s can be increased for improved accuracy once the desired convergence has been reached using a smaller value of s. We used $s = 1, 2, 5, 10$ rad^{-2}, with each cycle iterated 50 steps. The final step used a pseudo-random number based on the optimized probabilistic association functions in order to associate each conformation with a single cluster.

References

1. Hornak V, Abel R, Okur A, Strockbine B, Roitberg A, et al. (2006) Comparison of multiple amber force fields and development of improved protein backbone parameters. Proteins 65: 712 – 725.

2. Onufriev A, Bashford D, Case DA (2000) Modification of the generalized Born model suitable for macromolecules. J Phys Chem B 104: 3712-3720.

3. Onufriev A, Bashford D, Case DA (2004) Exploring protein native states and large-scale conformational changes with a modified generalized Born model. Proteins 55: 383-394.

4. Weiser J, Shenkin PS, Still WC (1999) Approximate Atomic Surfaces from Linear Combinations of Pairwise Overlaps (LCPO). J Comp Chem 20: 217-230.

5. Dominy BN, Brooks CL (1999) Development of a generalized born model parametrization for proteins and nucleic acids. J Phys Chem B 103: 3765 – 3773.

6. Moradi M, Babin V, Roland C, Sagui C (2010) A classical molecular dynamics investigation of the free energy and structure of short polyproline conformers. J Chem Phys 133: 125104.

7. Zimmerman SS, Pottle MS, Némethy G, Scheraga HA (1977) Conformational analysis of the 20 naturally occurring amino acid residues using ecepp. Macromolecules 10: 1-9.

8. Ramachandran GN, Ramakrishnan C, Sasisekharan V (1963) Stereochemistry of polypeptide chain configurations. J Mol Biol 7: 95 – 99.

9. Buhmann JM (1998) Stochastic algorithms for exploratory data analysis: Data clustering and data visualization. In: Learning in Graphical Models. Kluwer, pp. 405–420.

10. Dempster AP, Laird NM, Rubin D (1977) Maximum likelihood from incomplete data via the em algorithm. J Roy Statist Soc Ser B 39: 1 – 38.

11. MacQueen J (1967) Some methods for classification and analysis of multivariate observations. In: Proceedings of the Fifth Berkeley Symposium on Mathematical Statistics and Probability, Volume 1: Statistics. Univ. of Calif. Press, pp. 281–297.
